# Colchicine for the Treatment of Cardiac Injury in Hospitalized Patients With Coronavirus Disease-19

**DOI:** 10.3389/fcvm.2022.876718

**Published:** 2022-06-17

**Authors:** Amir Rabbani, Asim Rafique, Xiaoyan Wang, Danielle Campbell, Daniel Wang, Nicholas Brownell, Kenia Capdevilla, Victoria Garabedian, Sandra Chaparro, Raul Herrera, Rushi V. Parikh, Reza Ardehali

**Affiliations:** ^1^Division of Cardiology, University of California, Los Angeles, Los Angeles, CA, United States; ^2^Division of General Internal Medicine and Health Services Research, University of California, Los Angeles, Los Angeles, CA, United States; ^3^Miami Cardiac and Vascular Institute, Baptist Health South Florida, Miami, FL, United States

**Keywords:** COVID-19, colchicine, cardiac injury, myocarditis, inflammasome

## Abstract

**Introduction:**

The impact of colchicine on hospitalized patients with Coronavirus disease-19 (COVID-19) related cardiac injury is unknown.

**Materials and Methods:**

In this multicenter randomized controlled open-label clinical trial, we randomized hospitalized adult patients with documented COVID-19 and evidence of cardiac injury in a 1:1 ratio to either colchicine 0.6 mg po twice daily for 30 days plus standard of care or standard of care alone. Cardiac injury was defined as elevated cardiac biomarkers, new arrhythmia, new/worsened left ventricular dysfunction, or new pericardial effusion. The primary endpoint was the composite of all-cause mortality, need for mechanical ventilation, or need for mechanical circulatory support (MCS) at 90 days. Key secondary endpoints included the individual components of the primary endpoint and change in and at least 2-grade reduction in the World Health Organization (WHO) Ordinal Scale at 30 days. The trial is registered with clinicaltrials.gov (NCT04355143).

**Results:**

We enrolled 93 patients, 48 patients in the colchicine arm and 45 in the control arm. There was no significant difference in the primary outcome between the colchicine and control arms (19 vs. 15%, *p* = 0.78), nor in the individual components of all-cause mortality (17 vs. 15%, *p* = 1.0) and need for mechanical ventilation (8 vs. 5%, *p* = 0.68); no patients in either group required MCS. The change in (−1.8 ± 2.4 vs. −1.2 ± 2.0, *p* = 0.12) and at least 2-grade reduction (75 vs. 75%, *p* = 1.0) in the WHO ordinal scale was also similar between groups.

**Conclusion:**

Patients hospitalized with COVID-19 and evidence of cardiac injury did not benefit from colchicine therapy.

## Introduction

Coronavirus disease-19 (COVID-19), caused by the severe acute respiratory syndrome coronavirus 2 (SARS-CoV-2), has infected over 250 million patients and resulted in over 5 million deaths worldwide since December 2019. Cardiac injury in the setting of COVID-19 as defined by elevated cardiac biomarkers, arrhythmias, and/or structural abnormalities including ventricular dysfunction or pericardial effusion is common and has been reported in 16.1–62.3% of cases (1–7). Among patients with elevated cardiac biomarkers, mortality rates are approximately 30% in those without underlying cardiovascular disease (CVD) and up to 70% in those with underlying CVD (8–15). While several mechanisms have been postulated for how SARS-CoV-2 may damage the heart, it is plausible that the indirect injury from innate, cellular, or humoral immune responses including “cytokine storm” may play a pivotal role (16–20). To date, remdesivir has been approved by the FDA for hospitalized patients with severe pneumonia and several other agents have also been given Emergency Use Authorization. However, there are no FDA approved therapies specifically for the treatment of COVID-19 related cardiac injury (21).

Colchicine is a microtubule polymerization inhibitor and an inhibitor of interleukins 1 and 6, granulocyte macrophage colony stimulating factor (GM-CSF), and the nucleotide-binding oligomerization leucine-rich repeat and pyrin domain (NLRP3) inflammasome, making it a potent anti-inflammatory agent (22, 23). Its benefit in other inflammatory-based cardiovascular conditions has been established, including the treatment of acute and recurrent pericarditis, prevention of post-cardiotomy syndrome, and reduction of major adverse cardiovascular events after acute myocardial infarction (24–28). Although dose adjustments are required for certain comorbidities and concomitant drugs, colchicine is safe, cost-effective, widely available, and orally administered, making it an attractive potential therapeutic option for patients with COVID-19 (29–31).

Considering the prevalence of cardiac injury in COVID-19 patients and the associated high mortality rate among these patients, the need for effective treatment is critical, however, the impact of colchicine specifically among hospitalized COVID-19 patients with evidence of cardiac injury has not been described. In the present multicenter open-label RCT entitled Colchicine for the Treatment of Cardiac Injury in Hospitalized Patients with COVID-19 (COLHEART-19), we sought to determine if colchicine improved clinical outcomes in this key high-risk population.

## Materials and Methods

### Study Design

In this pilot multicenter open-label RCT, hospitalized adult patients with documented COVID-19 and evidence of cardiac injury were randomized in a 1:1 ratio to either colchicine 0.6 mg po twice daily for 30 days plus current standard of care or current standard of care alone. An adaptive trial design allowed for patients in either arm to be co-enrolled in other investigational therapeutic trials for COVID-19. Standard of care was defined as the current background treatment of COVID-19 at each institution, allowing for dynamic changes in therapy based on emerging research and experience during the rapidly evolving pandemic. The primary trial site was the University of California, Los Angeles (UCLA), with the Miami Cardiac and Vascular Institute (MCVI) at Baptist Health South Florida serving as a secondary site. The trial biostatistician (XW) was blinded to patient-level data and provided the randomization sequence and assignment using a permuted block design with varying block sizes between 2, 4, and 6 (SAS Version 9.4). All patients provided informed consent prior to enrollment. The trial protocol was approved by the institutional review boards at UCLA and MCVI; the trial was registered with clinicaltrials.gov (NCT04355143).

### Study Population

Adult patients (≥18 years) hospitalized with SARS-CoV-2 infection were eligible for COLHEART-19 if they had any of the following objective markers of cardiac injury: (1) elevated troponin, (2) newly elevated B-type natriuretic peptide (BNP), (3) new ischemic or arrhythmogenic changes on electrocardiogram (ECG) or telemetry, or (4) new reduction in left ventricular ejection fraction (LVEF) or new pericardial effusion on transthoracic echocardiogram (TTE). Exclusion criteria included severe hematologic or neuromuscular disorders, severe renal impairment with concomitant hepatic impairment, co-administration of CYP3A4 and P-glycoprotein transport system inhibitors, concurrent use of strong CYP3A4 or P-glycoprotein transport system inhibitors in patients with renal or hepatic impairment, pregnancy, breastfeeding mothers, or women of childbearing age unable to take adequate contraception. Additionally, patients taking colchicine for other indications (e.g., gout) or those already requiring mechanical ventilation or mechanical circulatory support (MCS) were ineligible for enrollment.

### Study Procedures

All patients underwent laboratory testing for cardiac biomarkers (troponin, BNP) and inflammatory biomarkers [C-reactive protein (CRP) and D-Dimer] as well as ECG and TTE on the day of enrollment if not already performed as part of their clinical care. Serial cardiac and inflammatory biomarkers were obtained on days 3 and 7 among those still hospitalized. Of note, to accommodate hospital logistics and workflow, the aforementioned tests were allowed to be collected within ±1 day of the study-assigned collection day.

Patients in both arms of the trial received the current standard of care treatment for COVID-19 per institutional protocols generated by local infectious disease and pulmonary/critical care medicine experts, though the final decision for treatment strategy was at the discretion of the care team. Those patients randomized to the colchicine arm received 0.6 mg twice daily for 30 days. Dose adjustments were made for gastrointestinal intolerance, co-morbidities such as chronic kidney disease, and drug-drug interactions. Additionally, all patients were eligible to be concurrently enrolled in other COVID-19 clinical trials. Telephone follow-up to evaluate symptoms was performed at 30 days, and telephone as well as electronic medical record follow-up to assess clinical outcomes was completed at 90 days.

### Endpoints

The primary endpoint was the composite of all-cause mortality, need for mechanical ventilation, or need for MCS at 90 days. Secondary endpoints included the individual components of the primary endpoint, time to the primary endpoint, change in the World Health Organization (WHO) R&D Blueprint Ordinal Scale at 30 days, and at least 2-grade reduction (i.e., clinical improvement) in the WHO Ordinal Scale at 30 days, re-hospitalization at 90 days, peak and maximum change (delta) in cardiac and inflammatory biomarkers (i.e., troponin, BNP, CRP, D-dimer), and length of hospital stay. Ordinal scales for clinical improvement have been used in prior COVID-19 therapeutic trials and consists of an 8-grade scale ranging from ambulatory without limitation of activities to death (32).

### Statistical Analysis

Descriptive statistics such as frequency and percentage were summarized for baseline demographic and clinical characteristics. For each study arm, the proportion of patients achieving the primary composite endpoint at 90 days was calculated, along with the 95% exact confidence interval (CI). Risk difference and the corresponding 95% CI between study arms were also estimated. Fisher’s exact test was used to compare the proportion of patients achieving the composite event between two study arms. Similar analyses were performed on the individual components (secondary endpoints) of the primary endpoint. For the secondary endpoint of time to the primary end point, methodologies used for time-to-event data were adopted. Log-rank test was used to compare the event curves between the study arms. Hazard ratios (HR) and the corresponding 95% CIs were obtained *via* a Cox proportional hazards regression model. For each of the other secondary endpoints, summary statistics such as mean, median, standard deviation, minimum, maximum were calculated and reported. Wilcoxon rank rum test was used to compare the two study arms. The analyses for the primary endpoint and the secondary endpoints were performed on the intent-to-treat (ITT) population. All analyses were run using complete cases; in the rare event of missing data, patients were excluded from analyses. All statistical tests were two-sided, with an alpha level of 0.05 as the cut-off for statistical significance. Statistical analyses were carried out using statistical software SAS Version 9.4.

## Results

### Baseline Characteristics

**FIGURE 1:**
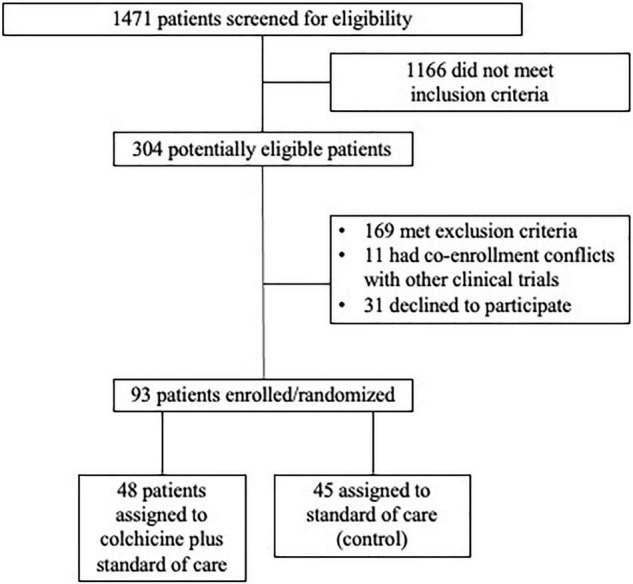
Study design.

Between May 5, 2020 and March 11, 2021, 93 hospitalized patients with COVID-19 were enrolled into the COLHEART-19 trial. The majority (85%) of patients were enrolled within the first 3 days of hospitalization and likely all were located on the hospital floor/wards at the time of enrollment [data regarding enrollment location—floor/wards vs. intensive care unit (ICU) was not captured, though mechanical ventilation and MCS which require ICU-level care were exclusion criteria]. Forty-eight patients were randomly assigned to colchicine plus standard of care (colchicine arm), while 45 patients were randomized to the standard of care (control arm) (Figure 1).

Baseline characteristics are summarized in Table 1. Of note, despite randomization, patients in the colchicine arm were more likely to be male (81 vs. 53%, *p* = 0.007) and had higher rates of hyperlipidemia (73 vs. 51%, *p* = 0.03) and chronic kidney disease (38 vs. 18%, *p* = 0.03) compared with those in the control arm. Otherwise, the two arms were largely balanced in their baseline demographic and clinical characteristics as well as COVID-19 related medications (Table 1).

**TABLE 1:** Baseline patient characteristics*.

	Colchicine *N* = 48	Control *N* = 45	*P-value*
**Demographics**			
Age (years)	71.2 ± 17	71.5 ± 19.5	0.86
Sex (male)	39 (81)	24 (53)	0.01
Race			0.57
White	28 (58)	21 (47)	
Black	2 (4)	4 (9)	
Other	18 (37)	20 (45)	
**Clinical risk factors**			
Hypertension	39 (83)	34 (76)	0.35
Hyperlipidemia	35 (73)	23 (51)	0.03
Diabetes	19 (40)	16 (36)	0.69
Tobacco use (current or former)	14 (29)	19 (42)	0.41
Cerebrovascular disease	4 (8)	8 (18)	0.18
Chronic kidney disease	18 (38)	8 (18)	0.03
COPD^†^	5 (10)	5 (11)	0.91
Other lung disease	5 (10)	5 (11)	0.91
Body mass index	29.6 ± 6.9	28.4 ± 8.0	0.24
**COVID-19^**†**^ therapeutics**			
Steroids	24 (56)	34 (72)	0.10
Remdesivir	36 (75)	29 (64)	0.37
Convalescent plasma	6 (13)	7 (16)	0.77
Hydroxychloroquine	1 (2)	0 (0)	1
Leronlimab	7 (15)	2 (4)	0.16
Gimsilumab	1 (2)	2 (4)	0.06
Anticoagulation			0.26
VTE^†^ prophylaxis dose	26 (54)	32 (71)	
Therapeutic dose	18 (38)	10 (22)	

*Data are presented as mean ± standard deviation or numbers (percentage) as appropriate.

*^†^COPD, chronic obstructive pulmonary disease; COVID-19, coronavirus disease-19 (COVID-19); VTE, venous thromboembolism.*

### Colchicine Data

Among the colchicine arm, 28 (58%) patients received the standard dose of 0.6 mg bid, while the remainder had the initial dose adjusted based on comorbidities and/or concomitant pharmacotherapy. Four (8%) patients underwent dose adjustments during the study period. With respect to adverse events related to colchicine, 7 (15%) patients experienced side effects that were classified as mild per protocol; no moderate or serious adverse events occurred. Of note, 4 of these patients terminated their courses of colchicine early due to the side effects. No patients in the control arm reported any side effects.

### Outcomes

#### Primary Endpoint

**FIGURE 2:**
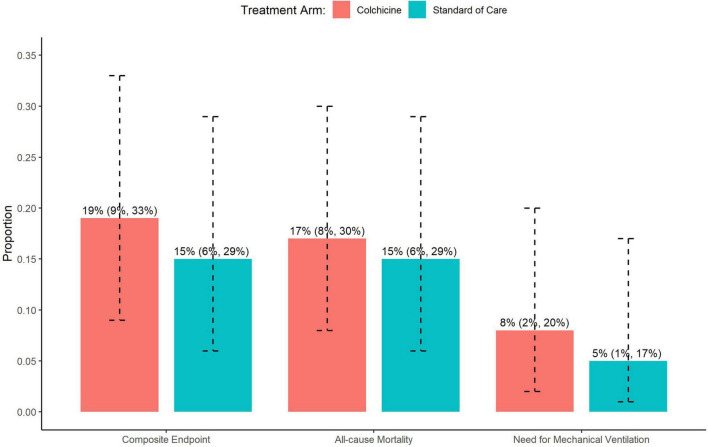
Impact of colchicine on the primary composite endpoint, and individual components of the primary composite endpoint including all-cause mortality and need for mechanical ventilation.

Overall, 96% of patients completed 90-day follow-up (4 patients in control arm and none in the colchicine arm were lost to follow-up). The proportion of patients who met the primary composite endpoint of all-cause mortality, need for mechanical ventilation, or need for MCS at 90 days was similar between the colchicine and standard of care arms (19 vs. 15%; *p* = 0.78) (Figure 2). These data remained statistically non-significant in a sensitivity analysis adjusting for the imbalance of gender, hyperlipidemia, and chronic kidney disease between groups (Table 2).

**TABLE 2:** Adjusted primary and individual component secondary endpoint data*.

	Colchicine *N* = 48	Control *N* = 45	Adjusted proportion difference
Primary endpoint (Composite of all-cause mortality, need for mechanical ventilation, or need for MCS at 90 days)	9 (19)	6 (15)	0%, 95% CI: −17%–18%, *p* = 0.96
All-cause mortality	8 (17)	6 (15)	1%, 95% CI: −18%–16%, *p* = 0.91
Need for mechanical ventilation	4 (8)	2 (5)	5%, 95% CI: −10%–20%, *p* = 0.51
Need for MCS^†^	0 (0)	0 (0)	0%

*Data are presented as numbers (percentage).

*^†^CI, confidence interval; MCS, mechanical circulatory support.*

#### Secondary Endpoints

**FIGURE 3:**
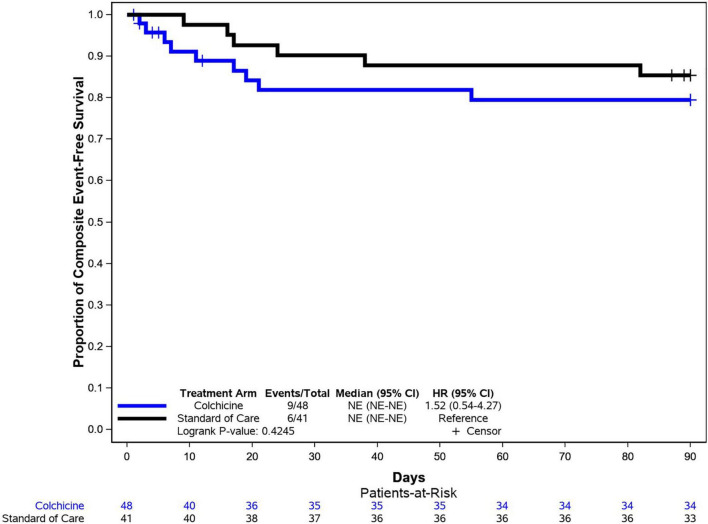
Kaplan–Meier analysis for the primary endpoint. Kaplan–Meier analysis demonstrated similar rates of event-free survival (composite of all-cause mortality, need for mechanical ventilation, or need for mechanical circulatory support) among patients in the colchicine and control arms.

There were no significant differences between the colchicine and control arms with respect to the individual components of the primary composite endpoint (Figure 2). Notably, the rate of mechanical ventilation was low in both groups (8 vs. 5%), and no patients in either group required MCS. The time to primary endpoint was not significantly different between the 2 arms (HR = 1.52, 95% CI: 0.57–4.27, *p* = 0.42) (Figure 3). Additionally, hospital length of stay (10.7 vs. 8.8 days, *p* = 0.20) and re-hospitalization rates at 90 days (15 vs. 22%, *p* = 0.37) were similar in the colchicine and control arms.

Patients in the colchicine arm had higher mean WHO ordinal scale scores at baseline compared with those in the control arm (4.2 ± 0.7 vs. 3.9 ± 0.8, *p* = 0.07). At 30 days, however, the change/reduction from baseline was similar between the colchicine and control arms (−1.8 ± 2.4 vs. −1.2 ± 2.0, *p* = 0.12), and there was no between-group difference in at least 2-grade reduction (75 vs. 75%, *p* = 1.0).

We also observed no significant difference in peak or delta troponin and BNP levels between the colchicine and control arms (Table 3). Patients in the colchicine arm did have significantly higher peak CRP levels (13.6 ± 8.9 vs. 9.8 ± 7.8, *p* = 0.03), though delta CRP as well as peak and delta D-dimer levels were similar between the 2 arms (Table 3).

**TABLE 3:** Cardiac and inflammatory biomarkers*.

	Colchicine	Control	*p*-value
**Troponin (ng/mL)**			
Baseline	0.1 ± 0.1	0.10 ± 0.5	0.37
Peak	0.2 ± 0.4	0.1 ± 0.5	0.16
Delta^†^	0.1 ± 0.3	0	0.06
**BNP^‡^ (pg/mL)**			
Baseline	503 ± 765	389 ± 798	0.42
Peak	611 ± 796	514 ± 817	0.42
Delta^†^	108 ± 218	134 ± 241	0.85
**CRP^‡^ (mg/dL)**			
Baseline	9.9 ± 7.3	7.2 ± 5.7	0.09
Peak	13.6 ± 8.9	9.8 ± 7.8	0.03
Delta^†^	3.9 ± 7.2	2.6 ± 4.1	0.57
**D-Dimer (μ g/mL)**			
Baseline	493 ± 854	669 ± 1101	0.92
Peak	639 ± 1112	861 ± 1564	0.95
Delta^†^	145 ± 353	212 ± 604	0.19

*Data are presented as mean ± standard deviation.

*^†^Delta is defined as the difference between peak and baseline values.*

*^‡^BNP, B-type natriuretic peptide; CRP, C-reactive protein.*

## Discussion

The principal finding of the multicenter randomized controlled open-label COLHEART-19 clinical trial is that colchicine did not reduce the primary endpoint of all-cause mortality, need for mechanical ventilation, or need for MCS at 90 days in hospitalized adult patients with COVID-19 and evidence of cardiac injury. The lack of benefit of colchicine extended across multiple key secondary endpoints including the individual components of the composite primary endpoint, change in and at least 2-grade reduction in the WHO Ordinal Scale at 30 days, and re-hospitalization at 90 days. To the best of our knowledge, COLHEART-19 is the first trial to specifically evaluate the impact of colchicine in hospitalized COVID-19 patients with manifestations of cardiac injury, and in doing so, adds to the growing body of evidence of its limited role in the treatment of COVID-19.

Multiple studies have previously suggested that colchicine may be an attractive therapeutic agent to treat patients presenting with COVID-19 given its unique anti-inflammatory properties, relative lack of serious side effects, and wide availability (30, 33–41). To date, several RCTs assessing the effect of colchicine on outcomes have been conducted in varying COVID-19 populations. Among hospitalized patients with COVID-19, the GRECCO-19 trial did not show a mortality benefit with colchicine administration, although there was a statistically significant decrease in 2-grade reduction in the WHO Ordinal Scale (42). Similarly, the larger RECOVERY trial, which randomized 11,162 hospitalized COVID-19 patients to colchicine vs. standard of care, also did not show a difference in the primary endpoint of 28-day mortality (risk ratio = 1.02, 95% CI: 0.94–1.11, *p* = 0.63) (43). Of note, several meta-analyses have suggested potential benefit for colchicine in hospitalized patients with COVID-19 with signals toward lower mortality, but these included mainly observational studies in addition to RCTs. Among outpatients with COVID-19, the recent COLCORONA trial randomized 4,488 patients to colchicine vs. placebo. COLCORONA failed to demonstrate an improvement in the primary endpoint of mortality or hospital admission (OR = 0.79, 95% CI: 0.61–1.03, *p* = 0.81). Interestingly, the authors made the observation that if only PCR-confirmed COVID-19 patients were included, there was a statistically significant improvement in the primary endpoint (OR = 0.75, 95% CI: 0.57–0.99, *p* = 0.042) (44).

In contrast to these prior RCTs, our COLHEART-19 trial is unique in that we focused on hospitalized COVID-19 patients with evidence of cardiac injury, a high-risk subgroup that theoretically would benefit the most from colchicine therapy. We hypothesized that treating these patients early during their hospitalization with colchicine would blunt the cytokine storm and improve short-term clinical outcomes. However, our results mirror those of the negative GRECCO-19, RECOVERY, and COLCORONA trials in that colchicine does not appear to be provide a benefit across multiple clinical and laboratory endpoints in hospitalized patients with COVID-19 and cardiac injury.

Our study had key limitations worth considering. First, our sample size did not reach our pre-specified sample size of 150 patients due to slower than expected enrollment at our institutions and hence is underpowered and precluded meaningful subgroup analyses. However, given the lack of a positive trend in the colchicine arm, it seems unlikely that a larger sample size would have changed the outcome of the trial. Second, the study had an open-label design. This open-label structure was specifically chosen to allow for co-enrollment into additional COVID-19 based therapeutic clinical trials at our institutions. Notably, the colchicine and control arms were balanced with respect to other COVID-19 investigational therapies, and we adjusted for this in our analyses. Third, the standard of care therapy in the control arm had significant variability owing to the rapidly evolving treatment of COVID-19 over the course of the study period as new research data and experience emerged; this introduces unmeasured confounding, though it also reflects real-world practice during a dynamic pandemic. Fourth, we elected for a short 30-day course of colchicine to treat acute COVID-19, though a longer course may have had a more appreciable impact on long-term outcomes. Fifth, we did not obtain follow-up imaging data (echocardiography or cardiac magnetic resonance imaging); although this would have provided additional valuable information, logistical and budgetary concerns precluded its inclusion in the protocol. Sixth, although troponin is an objective marker of cardiac injury, its heightened sensitivity may led to inclusion of patients without clinically significant COVID-19 cardiac involvement. Finally, our primary endpoint necessitated excluding hospitalized patients who immediately required intubation or MCS; however, we hypothesized that colchicine would have less of an impact in these critically ill patients whose inflammatory burden was already exceedingly high.

## Conclusion

In this multicenter open-label RCT, colchicine administration in hospitalized adult patients with COVID-19 and evidence of cardiac injury did not provide a benefit across multiple clinical and laboratory endpoints compared with standard of care. These findings are in agreement with other recent RCTs that similarly have not shown benefit of colchicine in COVID-19.

## Data Availability Statement

The raw data supporting the conclusions of this article will be made available by the authors, without undue reservation.

## Ethics Statement

The studies involving human participants were reviewed and approved by UCLA Institutional Review Board (IRB). The patients/participants provided their written informed consent to participate in this study.

## Author Contributions

AmR, AsR, RP, and RA had full access to all of the data in the study, took responsibility for the integrity of the data and the accuracy of the data analysis, and contributed to administrative, technical, or material support and supervision. AmR, XW, DW, AsR, RP, and RA drafted the manuscript. XW contributed to statistical analysis. All authors contributed to acquisition, analysis, or interpretation of data, contributed to the article, and approved the submitted version.

## Conflict of Interest

RP reports unrelated research support from the American Heart Association, Janssen, Infraredx, and Abbott Vascular; consulting fees from Abbott Vascular; serving on advisory board of HeartCloud, DocVocate, and Stallion Cardio. The remaining authors declare that the research was conducted in the absence of any commercial or financial relationships that could be construed as a potential conflict of interest.

## Publisher’s Note

All claims expressed in this article are solely those of the authors and do not necessarily represent those of their affiliated organizations, or those of the publisher, the editors and the reviewers. Any product that may be evaluated in this article, or claim that may be made by its manufacturer, is not guaranteed or endorsed by the publisher.
